# Editorial: Addressing the maternity needs of newly settled migrant mothers

**DOI:** 10.3389/fgwh.2026.1880736

**Published:** 2026-06-10

**Authors:** Mabel Leng Sim Lie, Alena Pařízková, Melanie Haith-Cooper

**Affiliations:** 1 Population Health Sciences Institute, Newcastle University, Newcastle upon Tyne, United Kingdom; 2 Department of Sociology and Social Work, University of West Bohemia, Pilsen, Czechia; 3 Research Institute for Health and Social Care, University of Bradford, Bradford, United Kingdom

**Keywords:** maternal health disparities, perinatal health, migrant mothers, maternity services, culturally responsive programmes

Statistical evidence and reviews describing disparities in maternal healthcare faced by migrant women have been building. At the population level, they face higher risks of stillbirth, perinatal and neonatal mortality (1), including higher odds of emergency caesarean birth, low Apgar scores, food insecurity, perinatal depression/anxiety, and intimate partner violence. Predictably, lacking understanding of western healthcare systems and language proficiency were the most frequently reported impeding factors affecting prenatal healthcare utilization (2). Gaps in evaluations of impact among a range of interventions have been identified, in particular among refugee and asylum-seeking women (3). Nevertheless, satisfaction in maternity care during resettlement has also been reported in some groups (4).

Examples of research in service provision include the relational care between midwives and mothers (5), a specialist migrant maternity service for asylum-seekers in their initial accommodation (6), a training course for midwives plus leaflet and mobile app (7) and group antenatal care (8). Interventions to address cultural awareness include employing a transcultural approach (9), specialized cross-cultural workers (10), an intercultural interpreting service (11) and culturally sensitive perinatal mental health care (12). The UK NHS Race and Health Observatory (13) review aimed to identify policies to address maternal health inequalities. Interventions successfully evaluated were largely based on service provider practice models, whereas those that involved link or liaison workers reportedly have not shown clear evidence of positive impact.

The diversity of women's maternity experiences has increased due to the surge in migration and displacement from wars and conflict, and insecurity across the globe. Their accounts can be found in studies of women from South Asia (14) and Africa (15), host countries of Canada (16), Australia (17), Switzerland (18), UK (19) and across Europe (20). Arguments for women's self-efficacy and better health literacy highlighted the need to understand preferences for participation in maternal care provision (21). The values women hold based on their sociocultural contexts may also come into conflict with those held by maternity service providers (16). Nevertheless, available technology such as multi-language videos (22) and pregnancy apps (23, 24) have been employed to facilitate communication. Mothers' reported fear of professionals (25) raised the need for dedicated staff to bridge the divides between mothers and maternity service providers, such as through employing health navigators (26), building women's confidence and trust in maternity services. This Frontiers special issue seeks to fill existing and growing knowledge gaps and add to this global evidence base.

Country-based contextual factors influence the provision, uptake and experience of maternity services, as demonstrated in the paper by Beesley and Lowe, reporting on the development of the “Nurture Together” initiative in Glasgow. Migrant mothers with precarious immigration statuses living in fear, isolation and discrimination in the UK's “hostile environment” were shown to benefit from the programme delivered by trained birth companions.

The significance of understanding parental beliefs about infant mortality is brought out in the paper on the Hispanic community in Indiana by Place et al., because of how they can influence behaviour during the antenatal period. This provides input for ways to promote perinatal care to pregnant Hispanic mothers, to overcome barriers to prenatal care reported in the first trimester.

Participatory workshops using a co-creation approach is featured in the article by Lie and Claisse, focusing on Arabic-speaking migrant mothers. They contributed text and drawings in an antenatal care community resource in the form of a letter that was digitally animated and recorded in English and Arabic by two workshop participants. QR codes in the resource provided links to information support for a newly arrived Arabic-speaking mother. The article offers insights into creative research methods in engagement with migrant mothers.

Cultural and community workers (CCWs) in maternity and early childhood care in Sydney, Australia, provide valued services for migrant and refugee women. Rogers et al. show how CCWs offer support, help women navigate healthcare, facilitate new social connections, and create a space for sharing culture and experience.

Critical Race Theory (CRT) served as the primary research framework for Atembeshu and Lafauts study of Black migrant women from sub-Saharan Africa receiving maternal care in Belgium during the COVID-19 pandemic. The authors analyse how pandemic-related restrictions exacerbated systemic racism, stereotyping, and linguistic racism and show how pandemic restrictions increased communication barriers, served to exclude women from decision-making processes, and led to inadequate care.

Li analyses interpreting services in maternal care and the systemic barriers they face. She describes a “vicious cycle” behind persistent inadequacy. In her analysis, Li points out how limited interpreting options, the undervaluation of interpreters, and poor infrastructure for technical innovation interconnect and sustain the cycle.

All three articles mention the COVID-19 pandemic and show how related restrictions had a significant negative impact on women's experiences and support. These restrictions led to greater isolation, fewer services, including interpreting services, and greater communication barriers. This supports the argument about the system of care and institutions' blindness to vulnerable migrant women and refugees.

The articles in this collection draw on research from Australia, Belgium, and the United Kingdom, as well as community-centred initiatives in Glasgow and the United States. Throughout these varied national contexts, they identify persistent barriers—language exclusion, structural racism, inadequate interpreting services, and the compounding effects of pandemic restrictions. At the same time, they point to what works: dedicated bicultural workers, co-created community resources, culturally responsive programmes delivered by trained birth companions, and systemic analyses that expose the interrelated nature of service failures. Together, they offer both a critical diagnosis and practical directions for improving maternity care for migrant women. This contribution to the current evidence base hopefully encourages more innovative intervention development, evaluation and implementation able to address persistent disparities in migrant maternal health (27).
